# Kawasaki Disease-Management Strategies Given Symptoms Overlap to COVID-19: A Review

**DOI:** 10.31729/jnma.5698

**Published:** 2021-04-30

**Authors:** Linna Wang, Sheng Zhang, Ji Ma, Jing Ni, Juyan Wang, Xiaohong Li, Zhilong Mu, Wei Han, Gaitao He, Lei Ma, Jenifei Shah, Jay Shah, Fuyong Jiao

**Affiliations:** 1 Xi'an Medical University, Xi'an, 710068, China; 2 Children's Hospital of Shaanxi Provincial People's Hospital, 3^rd^ Affiliated Hospital of Medical College of Xi'an Jiao Tong University, Xi'an Shanxi, 710001, China; 3 Ruijin Hospital, Shanghai Jiao Tong University School of Medicine, Shanghai, China; 4 Patan Hospital, Patan Academy of Health Sciences, Kathmandu, Nepal

**Keywords:** *children*, *COVID-19*, *Kawasaki disease*

## Abstract

Kawasaki disease is an acute, self-limiting vasculitis in children. Early treatment is necessary to prevent cardiovascular complications. The acute phase of Kawasaki disease may present with hemodynamic instability. An association between viral respiratory infections and Kawasaki disease has been reported. Studies have shown that Kawasaki and Kawasaki-like disease may be associated with and have symptoms overlapping COVID-19. Children with COVID-19 may present as Kawasaki-like disease with pediatric inflammatory multisystem syndrome, or macrophage activation syndrome. Clinicians need to be aware of the early diagnosis and management of Kawasaki disease to prevent the development of coronary artery aneurysms. The symptoms overlap of multisystem inflammatory disease seen in COVID-19 adds to the difficulties in timely diagnosis and treatment. Children with Kawasaki disease require regular follow-up plans for coronary artery aneurysms. This adds to the difficulties during the changed environment of COVID-19 for control and prevention. Missed diagnosis and early treatment of Kawasaki disease with immunoglobulin and aspirin results in the development of coronary artery aneurysm in up to 25% of cases, with grave consequences. Here, we briefly review the management of typical and atypical Kawasaki disease which has symptoms overlapping with the multisystem inflammatory disease as seen in COVID-19.

## INTRODUCTION

Kawasaki disease (KD) reported in 1967 is an acute, self-limiting vasculitis of the medium caliber vessels in children.^1,2^ Coronavirus disease 2019 (COVID-19) is increasingly reported in children.^3,4^ There is a concern for the hyperinflammatory state of COVID-19 and KD in multisystem inflammatory syndrome in children (MIS-C) or pediatric inflammatory multisystem syndrome (PIMS).^5-8^ Kawasaki-like disease with cardiac involvement and macrophage activation syndrome (MAS) has been reported from COVID-19 affected areas.^9-11^ Acute stage of KD may present with hemodynamic instability of KD shock syndrome (KDSS).^12^

The novel coronavirus (2019-nCoV) pneumonia reported from Wuhan, China in December 2019, was later renamed COVID-19 by the WHO.^13^ Association between viral infections and CIVID-19 have been reported in KD children.^14,15^ Management of KD is complicated due to the overlap of COVID-19 and MIS-C.^5,7,9,16-19^

We briefly review the clinical features of KD and possible associations with COVID-19 in children and timely management to prevent cardiovascular complications.

## METHODS

We performed an extensive review of Kawasaki Disease (KD), and Kawasaki-like disease which have been increasingly reported in children in association with COVID-19, and posing difficulties in the timely management of KD. Search methodology included data repositories of 'PubMed, Google Scholar, Google, Web of Science 'till October 2020. The following keywords, alone or in combination, were used: children, Kawasaki Disease, and Kawasaki-like disease, epidemiology, treatment guidelines, SARS-CoV-2, coronavirus disease 2019, COVID-19' to search the relevant literature. Titles and abstracts were assessed for relevance based on the contents of the full text. Articles for Kawasaki Disease and Kawasaki-like disease with relevance to COVID-19 in children for its clinical presentation and management were reviewed. The information was assessed and summarized on 'how children with KD or Kawasaki-like disease can be best managed with early recognition, early treatment with intravenous infusion of immunoglobulin (IVIG) and aspirin; plus, regular follow-up with echocardiography to minimize the long-term cardiovascular complications while considering transmission risk of COVID-19.

In this review, we found that Kawasaki Disease and Kawasaki-like disease have been increasingly reported in children in association with COVID-19 and may delay the diagnosis of KD. Thus, prompt initiation of specific treatment with IVIG, aspirin, and regular follow-up with echocardiography is recommended to minimize the long-term cardiovascular complications, especially the development and progression of coronary artery aneurysm.

## DISCUSSION

### Discovery and development of KD

KD was reported in Japan in 1967 by Dr. Tomisaku Kawasaki in a descriptive analysis of clinical manifestations of the disease in 50 children.^1,2^ It is an acute illness due to vasculitis of small and medium-size arteries presenting with prolonged fever, conjunctivitis, rashes, lips and tongue congestion, erythema, and swelling of the hands and feet, and cervical adenopathy. The disease is a self-limiting but cardiovascular complication of myocarditis, pericardial effusion, and coronary artery aneurysm is seen in up to 1/4^th^ of children and can be prevented by early treatment using IVIG and aspirin within 10 days of illness to prevent the development of coronary artery aneurysm (CAA).^20-22^

The etiology and pathogenesis of KD remain unclear. All ethnicities are affected and there is an increasing trend of this disease. Delays in diagnosis and treatment have serious effects, especially in developing countries.^23-24^ In developed countries, KD has replaced rheumatic fever as the main cause of acquired heart disease in children.

Geographically northeast Asian countries including Japan, South Korea, and China, have 10 to 30 times higher incidence of KD among children <5 years, than those of the USA (17.5-20.8/100,000) and Europe (5-10/100,000).^25^ Japan tops the list with almost 1 in 100 children having the disease by the age of 5 years, and the lowest incidence is seen in sub-Saharan Africa. This points towards a possible genetic predisposition, and immunological factors associated with the development of KD.^26,27^

The development of coronary artery lesions is considered a severe form of the disease, and several scoring systems have been proposed based on the clinical presentations and findings of echocardiography.^26^ The classification of KD is still debated. The infectious disease specialists claim it to be an infection that presents with a classic immune response to an unidentified pathogen which localizes to the coronary arteries; while another favored theory describes the autoimmune reaction to a homologous antigen to the vascular wall; meanwhile, rheumatologists classify it as a systemic vasculitis; furthermore, immunologists stress on innate immune response in children.^28^

### Clinical features and diagnosis of KD

The classical presentation of Kawasaki disease is characterized by systemic vasculitis presenting with acute febrile illness and rashes in children. The KD is also known as Mucocutaneous Lymph Node Syndrome (MCLS). It has a worldwide distribution and is more common in Asian children. It is the 2nd most common vasculitis seen in children after Henoch Schonlein purpura.^29^

Young children under 5-years of age account for >80% of the cases, with a peak age of 18-24 months. Children <6 months and >5 years are less commonly affected, but they are more likely to develop coronary artery aneurysms (CAA).^29,30^ The CAA is reportedly the most common cause of acquired heart disease in children, especially in developed countries.^26,29-32^ Even after proper treatment, the cardiovascular sequelae occurs, and they may present, in decreasing frequency, with coronary dilatation (1.90%), aneurysms (0.78%), valvular lesions (0.29%), giant aneurysms (0.22%), coronary stenosis (0.03%), and myocardial infarction (0.02%).^26,30^ The epidemiological study of KD from Japan shows that there is an increase in trend incidence, which was reported to be 239 per 100 000 children aged 0 to 4 years (in 2010), and with a higher rate during winter to spring months.^30^

There is no specific test for the diagnosis of KD. The diagnosis includes fever lasting more than 5 days plus at least four of the five clinical manifestations. If coronary artery abnormalities are present, KD can be diagnosed with less than four of the five features.^31-36^

**A. Complete/typical Kawasaki disease:**

These children have a fever of ≥5 days plus ≥4 out of the following five clinical manifestations

1. **Conjunctivitis-** Bilateral, dry or non-purulent, painless, preferentially bulbar in distribution. It is seen within 3-4 days after the onset of the disease. The purulent discharge dissipates after heat remission.
2. **Cervical lymphadenopathy-** Acute non-suppurative cervical lymph nodes, commonly unilateral, tender, usually >1.5 cm, hard, without redness. The nodes appear at the beginning of the disease and decrease in size when the fever subsides.
3. **Polymorphous rashes-** Polymorphous exanthema, without vesicles, bullae, or crusts. This occurs in the first few days, involves the trunk and extremities. Rashes may have variable presentations such as urticarial, morbilliform, maculopapular, or resembling scarlet fever. The rash appears in the first week, and perianal skin redness or peeling-off subsequently follows.
4. **Lips and oral mucosa hyperemia, Strawberry tongue-** Intense hyperemia of lip leads to congestion, red, chapped, or cracked lips, and/or diffuse erythema of oropharynx. The Strawberry tongue often protrudes outside the mouth due to congestion.
5. **Hands and feet hyperemia, edema-** In the acute phase, there is hyperemia and painful edema of hands/palms and feet/soles that progress to desquamation in the convalescent stage. Membranous desquamation from fingertips, peeling at the junction of the nail and skin of finger and toe may present with a transverse groove in the finger and toenails, and in severe cases, peeling-off of nails may be seen in the convalescent phase. Perineal desquamation is frequently associated.

**B. Incomplete/atypical KD:**

Children who do not meet the above criteria and are suspected of incomplete/atypical KD i.e. child with fever ≥5 days plus two or three compatible clinical criteria, or fever ≥7 days without any other explanation.^31,34,36^ It is more frequent in the extremes of ages, among infants <1 year and >8 years.^24^ The diagnostic criteria are based on supportive laboratory findings and echocardiography. Children with incomplete/atypical KD require evaluation in a step-by-step algorithm.

### Clinical phases of KD

Based on distinct clinical characteristics of KD, it has 3-phases of clinical significance.^20^

1. Acute febrile phase: 1-2 weeks, with fever plus other acute features; myocarditis; pericardial effusion.
2. Subacute phase: until day 30 of illness, with fever resolution, possible persistence of conjunctival injection and irritability, desquamation of fingers and toes, thrombocytosis, coronary arteritis; risk of sudden death.
3. Convalescent phase: 6-8 weeks after the onset of illness, clinical signs of illness resolved; lasts until sedimentation rate normalizes.

### Role of echocardiography in KD

Echocardiography should be considered and repeated at 1-2 and 4-6 weeks after treatment.^31,34,35^ For echocardiogram positive, children should be treated within 10 days of onset of fever and beyond day 10 with clinical and laboratory signs (CRP, ESR) of ongoing inflammation.^31^ The echocardiogram is considered positive if any of the three conditions are met: z score of the left anterior descending (LAD) or right coronary artery (RCA) ≥2.5, coronary arteries meet Japanese Ministry of Health criteria for aneurysms, or ≥3 other suggestive features exist, including perivascular brightness, lack of tapering, decreased LV function, mitral regurgitation, pericardial effusion, or Z scores in LAD or RCA of 2-2.5.

### Grading of coronary artery aneurysm (CAA)

Quantitative measurement of coronary artery luminal dimensions and coronary artery aneurysm (CAA) is based on normalized as Z-scores adjusted for body surface area.^35,36^

1. No involvement: Always <2
2. Dilation only: 2 to ≤2.5; or if initially <2, a decreasein Z-score during follow-up ≥1
3. Small aneurysm: ≥2.5 to <5
4. Medium aneurysm: ≥5 to <10, andabsolutedimension <8 mm
5. Large or giant aneurysm: ≥10, orabsolutedimension ≥8 mm

### Management of KD in view of COVID-19 pandemic

Typically, Kawasaki disease is diagnosed based on clinical presentations as there is a lack of specific tests. Early recognition and treatment with intravenous immunoglobulin (IVIG) are necessary to reduce cardiovascular complications, especially the development of CAA.^33,37^

Even though KD is seen in all seasons, it peaks in winter and early summer, a season for viral respiratory illnesses.^32,38^ There have been reports from Europe and America that children with KD-like symptoms (fever, skin rash, conjunctivitis; oral mucosa changes with red fissured lips, strawberry tongue, hand or foot edema) along with cardiovascular complications (left ventricular dysfunction, myocarditis, pericarditis, valvular regurgitation, coronary arterial ectasia or aneurysm) also tested positive for COVD-19 in up to two-thirds of cases.^6,19^

The source of infection in children is mainly from patients infected with COVID-19 with or without symptoms. The transmission route is mainly through droplets and contact. The risk of fecal-oral transmission has been reported. Mother-to-child transmission has been reported in a patient diagnosed with COVID-19 showing nucleic acid test positive in the newborn 30 hours after birth.^39^

Protection of children and family from COVID-19 requires some modification to the general measures applied for adults, for example:

1. Hand hygiene is equally important for children. Younger children require assistance from parents or adults. Parents are required to wash their hands first, then help their children, and wash their hands again after helping them.
2. Wearing a face mask correctly is very important. The mask should completely cover the mouth and nose. Children over the age of 1 year need to wear a mask when going out. An N95 mask or a disposable surgical mask for children is recommended in high-risk areas. Parents should pay attention to children's breathing while wearing masks to avoid suffocation. Parents also need to wear masks correctly and wear masks themselves before helping children.
3. Personal hygiene should also be maintained with frequent showers, manicures, change of clothing, and keeping children clean and tidy to avoid contact with secretions.
4. Public gatherings should be avoided to minimize the risk of exposure to the source of infection. If it's necessary to go out, a mask should always be worn to minimize exposure to public transportations and elevators.

Similarly, protective measures for adult family members themselves and their children, for example:

1. Minimize visits to suspected or confirmed COVID-19 people, observe proper use of masks, and hand washing. Minimize outing activities and visitors in the family. When encountering confirmed COVID-19 or suspected person, strict isolation for at least 14 days, and avoid contact with children.
2. All family members should be aware and observe for the features of COVID-19 infection, i.e. fever, cough, chest tightness, shortness of breath, vomiting, diarrhea, fatigue, etc.
3. Ventilation and cleanliness of the home are necessary. Surfaces and objects which come in contact frequently, such as elevator buttons, door handles, light switches, TV remotes, mobile phones, should be cleaned and disinfected regularly.

### Treatment, monitoring, and follow-up of KD

The overlap in systematic inflammatory symptoms in children with KD and suspected COVID-19 poses new challenges to the diagnosis and treatment. Overlap of SARS-CoV-2 and KD in the form of multisystem inflammatory syndrome and an increase in the incidence of KD-like diseases after the outbreak of COVID-19 suggests the coronavirus may be associated with KD.^40^

**A. Screening of children with fever during COVID-19 pandemic:**

Triaging is important for children to diagnose KD promptly for early treatment. Implementation of a reasonable plan for the treatment of febrile children who may have KD requires the development of a triage plan for proper screening given the risk of COVID-19 transmission.^41^ Children with KD need regular follow up to review their cardiac ultrasound, and thus a reasonable plan should be formulated to minimize the effect of changing working environment in hospitals due to pandemic. Some of the useful approaches to minimize coronavirus transmission are - online appointments and consultation, treatment in the nearest local hospital, remote video consultations, inpatient diagnosis, and treatment in non-COVID hospitals.

**B. Treatment guidelines for KD:**

The early treatment of KD has a good recovery. Timely treatment with intravenous immunoglobulin (IVIG) is important to reduce cardiovascular complications.^33,37^ The IVIG 1-2 gm/kg body weight in a single infusion over 10-12 hours, and addition of aspirin (Acetylsalicylic acid, ASA) in moderate (30-50 mg/kg/day) dose until afebrile.^35,36^ Higher doses of IVIG favored mostly in North American countries, do not show added clinical efficacy over 1gm dose in most Asian countries, as shown in the Japanese guideline and meta-analysis.^36,42^ Methylprednisolone single-dose pulse therapy may be added, while a longer course of corticosteroid, IVIG, and aspirin may be required for high-risk, refractory acute KDs. The IVIG treatment is safe, but rare side effects like chills, anaphylactic reactions, hemolytic anemia, thrombocytopenia, acute renal failure, etc have been reported.^36^ While moderate-dose of aspirin is beneficial, the high dose (80-100 mg/kg/ day) has been associated with anemia and rarely Reye syndrome without benefit to disease outcomes.^43,44^ The findings of a recent Japanese study suggest that aspirin 50 mg/kg/day does not significantly improve the incidence of CAA compared with 30 mg/kg/day.^45^ In 10-20% of KD, there is persistent or recurrent fever, and these children require an additional second dose of IVIG, high-dose pulse, or a long course of steroids, infliximab, cyclosporine, and immunomodulatory monoclonal antibody.

Refractory KD are resistant to IVIG therapy, and these children possibly harbor resistant genes.^40,46^ A metanalysis has shown that initial administration of IVIG ≤4.0 days after the onset of symptoms, increased ESR and decreased hemoglobin and platelet counts, oral mucosa alterations, cervical lymphadenopathy, swelling of the extremities, and polymorphous rash are risk factors for IVIG-resistant KD.^47^ Coronary artery involvement is a severe form of the disease, and scoring systems have been proposed to stratify the risk factors and severity.^26^

Cyclophosphamide, methotrexate, and plasma exchange therapy have also been used in treatment-resistant cases of KD.^45^ Plasma exchange therapy has shown favorable outcomes.^48,49^ Vitamin D supplementation and breastfeeding have a protective effect on KD.^50^ Vitamin D deficiency is a risk factor for non-response to IVIG therapy.^51^

**C. Follow-up plan of KD:**

Children with newly diagnosed or re-diagnosed KD after initial management require regular follow-up to review their cardiac ultrasound, and thus a reasonable plan should be formulated to minimize the effect of changing the working environment in hospitals due to the COVID-19 pandemic.

Children with normal coronary arteries in the acute phase of Kawasaki disease undergo a comprehensive examination (physical, electrocardiogram, and echocardiography) during follow-up at 1, 3, 6 months, and 1 to 2 years after discharge. During the first three months review for the normalcy of erythrocyte sedimentation rate, C-reactive protein, and myocardial enzymes should be included. Coronary aneurysms occur in 15% to 25% of children with KD who have not been effectively treated. Long-term follow-up once every 6 to 12 months, and in case of any abnormality a monthly review, and possibly a coronary angiography is required. Coronary aneurysm disappears spontaneously 2 years after the disease, but often has abnormalities such as thickening of the wall and lessening of elasticity. Large aneurysms often do not completely disappear and may cause thrombosis or stenosis.^2,31,36^

The subset of patients with coronary artery aneurysms who have had KD treated in childhood or some missed cases of KD require lifelong follow-up due to the risk for ischemic events in adult life.^52^ This antecedent KD (missed cases), patients or parents may recall prolonged acute fever, rash, “bloodshot” eyes, and peeling of the fingers and toes during the convalescent phase and managed for various viral illnesses, like measles, scarlet fever, allergic reaction, etc. Computed tomography may show calcification of coronary arteries, and magnetic resonance imaging may reveal myocardial scarring and fibrosis.^52^

The question of 'lifelong follow up' for patients with KD should require consideration for the cost, anxiety to patients, and availability of resources.^52^ Studies show that a conservative approach of echocardiographic evaluation of the coronary arteries at initial presentation, and follow-up at 6-8 weeks and 6-12 months suffice in children who did not have or had only transient coronary artery involvement during the acute phase. Echocardiographic follow-up after the first year is of less value unless cardiac disease is _suspected._^26,35,53^

Lack of awareness and delay in management are seen in clinical practice. Such concern has been observed in most Latin American countries, and a high recurrence rate of 8.3%, including multiple recurrences of KD, has been reported from Brazil. The report also found long-term behavioral abnormalities presenting as irritability, aggressiveness, and learning deficit observed during follow-up.^24^ This contrasts with the low recurrence (2.4%) reported in a Swiss study of 30 years of KD.^53^ Similarly, the lack of awareness and a delay in diagnosis have been reported from India ^54-56^ and Nepal^57^ in Asia. The study from Nepal reported a low incidence of 0.1% i.e. 4 complete and 8 incomplete KD out of 11416 pediatric admissions for 5 years from a tertiary care teaching hospital.

Overall, a medium to long-term prognosis is good after the early treatment of KD. A subset of children with risk factors for poor outcomes, like the male gender, atypical age, and lack of IVIG therapy have higher cardiovascular complications and long-term sequelae.

## WAY FORWARD

Kawasaki disease is an acute, self-limiting vasculitis in children. The acute phase of Kawasaki disease may present with hemodynamic instability of Kawasaki disease shock syndrome. Children with COVID-19 may present as Kawasaki-like disease with the multisystem inflammatory syndrome. Clinicians need to be aware of early diagnosis and management of Kawasaki disease to prevent the development of coronary artery aneurysm. Kawasaki disease has an overlap of symptoms of multisystem inflammatory disease seen in COVID-19. Kawasaki disease requires regular follow-up for coronary artery aneurysm, adding to the difficulties for control and transmission of COVID-19. Missed diagnosis and lack of early treatment of Kawasaki disease have up to 25% chances of development of coronary artery aneurysm and cardiovascular complications.

## References

[1] Kawasaki T, Kosaki F, Okawa S, Shigematsu I, Yanagawa H (1974). A new infantile acute febrile mucocutaneous lymph node syndrome (MLNS) prevailing in Japan. Pediatrics.

[2] Kato H, Sugimura T, Akagi T, Sato N, Hashino K, Maeno Y (1996). Long-term consequences of Kawasaki disease. A 10- to 21-year follow-up study of 594 patients. Circulation.

[3] Gao Q, Liu J, Mu Z, Yan X, Shah JN, Jiao F (2020). Clinical profile of covid-19 in children and research progress on angiotensinconverting enzyme 2: A Mini-review. JNMA J Nepal Med Assoc.

[4] Dong Y, Mo X, Hu Y, Qi X, Jiang F, Jiang Z (2020). Epidemiology of COVID-19 among children in China. Pediatrics.

[5] Akca UK, Kesici S, Ozsurekci Y, Aykan HH, Batu ED, Atalay E (2020). Kawasaki-like disease in children with COVID-19. Rheumatol Int.

[6] Belhadjer Z, Meot M, Bajolle F, Khraiche D, Legendre A, Abakka S (2020). Acute heart failure in multisystem inflammatory syndrome in children (MIS-C) in the context of global SARS-CoV-2 pandemic. Circulation.

[7] Toubiana J, Poirault C, Corsia A, Bajolle F, Fourgeaud J, Angoulvant F (2020). Kawasaki-like multisystem inflammatory syndrome in children during the covid-19 pandemic in Paris, France: prospective observational study. BMJ.

[8] Harwood R, Allin B, Jones CE, Whittaker E, Ramnarayan P, Ramanan AV (2020). A national consensus management pathway for pediatric inflammatory multisystem syndrome temporally associated with COVID-19 (PIMS-TS): results of a national Delphi process. Lancet Child Adolesc Health.

[9] Verdoni L, Mazza A, Gervasoni A, Martelli L, Ruggeri M, Ciuffreda M (2020). An outbreak of severe Kawasaki-like disease at the Italian epicenter of the SARS-CoV-2 epidemic: an observational cohort study. Lancet.

[10] Panupattanapong S, Brooks EB (2020). New spectrum of COVID-19 manifestations in children: Kawasaki-like syndrome and hyperinflammatory response. Cleve Clin J Med.

[11] Wang W, Gong F, Zhu W, Fu S, Zhang Q (2015). Macrophage activation syndrome in Kawasaki disease: more common than we thought?. Semin Arthritis Rheum.

[12] Kanegaye JT, Wilder MS, Molkara D, Frazer JR, Pancheri J, Tremoulet AH (2009). Recognition of a Kawasaki disease shock syndrome. Pediatrics.

[13] Jin Y-H, Cai L, Cheng Z-S, Cheng H, Deng T, Fan Y-P (2020). A rapid advice guideline for the diagnosis and treatment of 2019 novel coronavirus (2019-nCoV) infected pneumonia (standard version). Mil Med Res.

[14] Turnier JL, Anderson MS, Heizer HR, Jone P-N, Glodé MP, Dominguez SR (2015). Concurrent Respiratory Viruses and Kawasaki Disease. Pediatrics.

[15] Chang L-Y, Lu C-Y, Shao P-L, Lee P-I, Lin M-T, Fan T-Y (2014). Viral infections associated with Kawasaki disease. J Formos Med Assoc.

[16] Pouletty M, Borocco C, Ouldali N, Caseris M, Basmaci R, Lachaume N (2020). Paediatric multisystem inflammatory syndrome temporally associated with SARS-CoV-2 mimicking Kawasaki disease (Kawa-COVID-19): a multicentre cohort. Ann Rheum Dis.

[17] Verdoni L, Mazza A, Gervasoni A, Martelli L, Ruggeri M, Ciuffreda M (2020). An outbreak of severe Kawasaki-like disease at the Italian epicentre of the SARS-CoV-2 epidemic: an observational cohort study. Lancet.

[18] Davies P, Evans C, Kanthimathinathan HK, Lillie J, Brierley J, Waters G (2020). Intensive care admissions of children with paediatric inflammatory multisystem syndrome temporally associated with SARS-CoV-2 (PIMS-TS) in the UK: a multicentre observational study. Lancet Child Adolesc Health.

[19] Whittaker E, Bamford A, Kenny J, Kaforou M, Jones CE, Shah P (2020). Clinical characteristics of 58 children with a pediatric inflammatory multisystem syndrome temporally associated with SARS-CoV-2. JAMA.

[20] Rowley AH, Shulman ST (1998). Kawasaki Syndrome. Clin Microbiol Rev.

[21] Burns JC, Glode MP (2004). Kawasaki syndrome. Lancet.

[22] Newburger JW, Takahashi M, Burns JC (2016). Kawasaki Disease. J Am Coll Cardiol.

[23] Singh S, Sharma A, Jiao F (2016). Kawasaki Disease: Issues in Diagnosis and Treatment--A Developing Country Perspective. Indian J Pediatr.

[24] Medeiros R, de Magalhaes C, Coutinho de Almeida F, Gandolfi L, Pratesi R, Ribeiro de M., Alves N, Selleski N (2020). Clinical manifestations of kawasaki disease at different age spectrum: a ten-year study. Medicina (Kaunas).

[25] Lin M-T, Wu M-H (2017). The global epidemiology of Kawasaki disease: Review and future perspectives. Glob Cardiol Sci Pract.

[26] JCS Joint Working Group. (2014). Guidelines for diagnosis and management of cardiovascular sequelae in Kawasaki disease (JCS 2013). Digest version. Circ J.

[27] Elakabawi K, Lin J, Jiao F, Guo N, Yuan Z (2020). Kawasaki Disease: Global Burden and Genetic Background. Cardiol Res.

[28] Marrani E, Burns JC, Cimaz R (2018). How Should We Classify Kawasaki Disease?. Front Immunol.

[29] The Royal Children's Hospital Melbourne. (2021). Clinical Practice Guidelines: Kawasaki disease [Internet].

[30] Nakamura Y, Yashiro M, Uehara R, Sadakane A, Tsuboi S, Aoyama Y (2012). Epidemiologic features of Kawasaki disease in Japan: results of the 2009-2010 nationwide survey. J Epidemiol.

[31] Newburger JW, Takahashi M, Gerber MA, Gewitz MH, Tani LY, Burns JC (2004). Diagnosis, treatment, and long-term management of kawasaki disease: a statement for health professionals from the committee on rheumatic fever, endocarditis, and kawasaki disease, council on cardiovascular disease in the young, american heart association. Pediatrics.

[32] McCrindle Brian W, Rowley Anne H, Newburger Jane W, Burns Jane C, Bolger Anne F, Michael Gewitz (2017). Diagnosis, treatment, and long-term management of kawasaki disease: a scientific statement for health professionals from the american heart association. Circulation.

[33] Yan F, Pan B, Sun H, Tian J, Li M (2019). Risk factors of coronary artery abnormality in children with kawasaki disease: a systematic review and meta-analysis. Front Pediatr.

[34] Yu JJ (2012). Diagnosis of incomplete Kawasaki disease. Korean J Pediatr.

[35] McCrindle BW, Rowley AH, Newburger JW, Burns JC, Bolger AF, Gewitz M (2017). Diagnosis, treatment, and long-term management of kawasaki disease: a scientific statement for health professionals from the american heart association. Circulation.

[36] Research Committee of the Japanese Society of Pediatric Cardiology, Cardiac Surgery Committee for Development of Guidelines for Medical Treatment of Acute Kawasaki Disease. (2014). Guidelines for medical treatment of acute kawasaki disease: report of the research committee of the japanese society of pediatric cardiology and cardiac surgery (2012 revised version). Pediatr Int.

[37] Choi J-W (2020). Can we get a clue for the etiology of Kawasaki disease in the COVID-19 pandemic?. Clin Exp Pediatr.

[38] Rowley AH, Shulman ST (2018). The epidemiology and pathogenesis of kawasaki disease. Front Pediatr.

[39] Jy Z, Jy Y, Jm Q (2020). Interpretations of "diagnosis and treatment protocol for novel coronavirus pneumonia (trial version 7)". Chin Med J (Engl).

[40] Zhang RL, Lo HH, Lei C, Ip N, Chen J, Law BY-K (2020). Current pharmacological intervention and development of targeting IVIG resistance in Kawasaki disease. Curr Opin Pharmacol.

[41] Ding H, Shi Z, Ruan Z, Cheng X, Li R, Zhang L (2020). Consideration of the management of pediatric fever clinics during the novel coronavirus pneumonia outbreak. Disaster Med Public Health Prep.

[42] Chen J, Ma B, Lin L-X, Xue Y-M (2012). Treatment of Kawasaki disease by different doses of immunoglobulin: a meta analysis of efficacy and safety. Transl Pediatr.

[43] Kuo H-C, Lo M-H, Hsieh K-S, Guo MM-H, Huang Y-H (2015). High-Dose Aspirin is associated with anemia and does not confer benefit to disease outcomes in kawasaki disease. PloS One.

[44] Kwon JE, Roh DE, Kim YH (2020). The impact of moderate-dose acetylsalicylic acid in the reduction of inflammatory cytokine and prevention of complication in acute phase of kawasaki disease: the benefit of moderate-dose acetylsalicylic acid. Child Basel Switz.

[45] Ito Y, Matsui T, Abe K, Honda T, Yasukawa K, Takanashi J-I (2020). Aspirin dose and treatment outcomes in kawasaki disease: a historical control study in japan. Front Pediatr.

[46] Duignan S, Doyle SL, McMahon CJ (2019). Refractory Kawasaki disease: diagnostic and management challenges. Pediatric Health Med Ther.

[47] Li X, Chen Y, Tang Y, Ding Y, Xu Q, Sun L (2018). Predictors of intravenous immunoglobulin-resistant Kawasaki disease in children: a meta-analysis of 4442 cases. Eur J Pediatr.

[48] Noguchi S, Saito J, Kudo T, Hashiba E, Hirota K (2018). Safety and efficacy of plasma exchange therapy for Kawasaki disease in children in intensive care unit: case series. J A Clin Rep.

[49] Hokosaki T, Mori M, Nishizawa T, Nakamura T, Imagawa T, Iwamoto M (2012). Long-term efficacy of plasma exchange treatment for refractory Kawasaki disease. Pediatr Int Off J Jpn Pediatr Soc.

[50] Meyer K, Volkmann A, Hufnagel M, Schachinger E, Klau S, Horstmann J (2019). Breastfeeding and vitamin d supplementation reduce the risk of kawasaki disease in a german population-based case-control study. BMC Pediatr.

[51] Jun JS, Jung YK, Lee DW (2017). Relationship between vitamin D levels and intravenous immunoglobulin resistance in Kawasaki disease. Korean J Pediatr.

[52] Gordon JB, Kahn AM, Burns JC (2009). When children with kawasaki disease grow up: myocardial and vascular complications in adulthood. J Am Coll Cardiol.

[53] Dajani AS, Taubert KA, Takahashi M, Bierman FZ, Freed MD, Ferrieri P (1994). Guidelines for long-term management of patients with kawasaki disease. report from the committee on rheumatic fever, endocarditis, and kawasaki disease, council on cardiovascular disease in the young, american heart association. Circulation.

[54] Balasubramanian S, Krishna MR, Dhanalakshmi K, Amperayani S, Ramanan AV (2012). Factors associated with delay in diagnosis of Kawasaki disease in India. Indian Pediatr.

[55] Jiao F, Jindal AK, Pandiarajan V, Khubchandani R, Kamath N, Sabui T (2017). The emergence of kawasaki disease in india and china. Glob Cardiol Sci Pract.

[56] Singh S, Kawasaki T (2016). Kawasaki disease in india, lessons learnt over the last 20 years. Indian Pediatr.

[57] Rajak K, Twayana AR, Shrestha R, Amatya P, Ghimire C (2019). Prevalence of kawasaki disease in a tertiary care hospital: a descriptive cross-sectional study. JNMA J Nepal Med Assoc.

